# Dermal Drug Delivery of Phytochemicals with Phenolic Structure via Lipid-Based Nanotechnologies

**DOI:** 10.3390/ph14090837

**Published:** 2021-08-24

**Authors:** Viliana Gugleva, Nadezhda Ivanova, Yoana Sotirova, Velichka Andonova

**Affiliations:** Department of Pharmaceutical Technologies, Faculty of Pharmacy, Medical University of Varna, 55 Marin Drinov Str., 9000 Varna, Bulgaria; nadejda.ivanova@mu-varna.bg (N.I.); Yoana.Sotirova@mu-varna.bg (Y.S.); Velichka.Andonova@mu-varna.bg (V.A.)

**Keywords:** biologically active compounds, dermal drug delivery, liposomes, nanoemulsions, nanostructured lipid carriers, polyphenols, phytophenols, solid lipid nanoparticles, skin permeation

## Abstract

Phenolic compounds are a large, heterogeneous group of secondary metabolites found in various plants and herbal substances. From the perspective of dermatology, the most important benefits for human health are their pharmacological effects on oxidation processes, inflammation, vascular pathology, immune response, precancerous and oncological lesions or formations, and microbial growth. Because the nature of phenolic compounds is designed to fit the phytochemical needs of plants and not the biopharmaceutical requirements for a specific route of delivery (dermal or other), their utilization in cutaneous formulations sets challenges to drug development. These are encountered often due to insufficient water solubility, high molecular weight and low permeation and/or high reactivity (inherent for the set of representatives) and subsequent chemical/photochemical instability and ionizability. The inclusion of phenolic phytochemicals in lipid-based nanocarriers (such as nanoemulsions, liposomes and solid lipid nanoparticles) is so far recognized as a strategic physico-chemical approach to improve their in situ stability and introduction to the skin barriers, with a view to enhance bioavailability and therapeutic potency. This current review is focused on recent advances and achievements in this area.

## 1. Introduction

Phenolics are a large group of secondary metabolites comprising one or more phenolic rings in their chemical composition [1]. The myriad structural variations determine an inherent diversity and heterogeneity in the group. The over 8000 identified representatives of herbal/vegetable origin differ in the number of phenolic rings and phenolic groups, the presence of other substitutes of the H-atom/s in the aromatic core, and the level of saturation/dehydration [2,3]. Subgroups are the simple phenols (phenolic acids, alcohols, and others), the flavonoids, anthraquinones, naphtoquinones, acetophenones, xanthones, stilbenes, tannins, phloroglucinols, and lignans [2,3]. Despite the structural variety, the majority of phenolics exhibit antioxidant, anti-inflammatory, and antimicrobial activity in vivo [4,5,6], to which they principally owe their therapeutical potential in the treatment of series of health disorders [7]. Furthermore, such a pharmacological profile justifies the increasing interest in the utilization of phenolic compounds in cosmetics for esthetic purposes (antiaging, antihyperpigmentation products, and others) [8,9,10,11]. From the clinical perspective of dermatology, the local or systemic application of phenolic compounds may contribute to the cure or prevention of many skin diseases. Among them are cancerous or precancerous conditions, acne vulgaris, allergies, rosacea, atopic dermatitis, psoriasis, vitiligo, wounds, and many more [12]. Widely explored members of the phytophenolics group in the therapy of dermatological problems include caffeic, ferulic, chlorogenic, coumaric and gallic acids, resveratrol, catechins, quercetin, rutin, kaempferol, curcumin, luteolin, hypericin, hyperforin [2,13,14,15,16]. Many other potent representatives, as well as herbal extracts rich in phenolic content, have fallen under the therapeutic focus of skin diseases/disorders. However, the main setbacks to the dermal delivery of phenolic compounds appear to be their chemical instability and potential discrepancy with the biopharmaceutical requirements for this route of application [17,18]. Dermal drug transport is dictated, to a large extent, by the physico-chemical particularities of the active ingredients. Extreme polarity or strict hydrophobicity, high molecular mass, the presence of ionizable functional groups and their dissociation at the physiological/pathophysiological pH of the skin layers are all prerequisites for limited cutaneous permeation of the drug [19,20]. Since one or more of them are intrinsic for the majority of phenolic compounds, they do not always represent the best candidates for dermal transport [21,22]. Another limitation is often set by insufficient chemical stability of particular representatives [23,24] (e.g., resveratrol [25,26,27], hypericin [28,29], hyperforin [30,31,32], quercetin [33,34,35], cathehin [36,37,38]), for which precise control over the selection of dermal vehicles and technological operations for drug introduction in preformulation stage is required. It is worth mentioning that physico-chemical properties, skin permeation, and chemical stability of phenolics are strongly affected by the presence of the glycoside part attached to the aglycone, and its type [39,40]. Most often, but not always, the phenolic aglycons are preferred for dermal delivery as a result of their higher permeability coefficient and skin deposition, unless pharmacological/toxicological reasons or stability considerations direct the choice of researchers in favor of a glycoside form [21,41,42,43].

The inclusion of active pharmaceutical ingredients in drug delivery systems is the contemporary approach to overcome problems such as poor solubility, stability, and permeation [44,45,46]. Indisputably, lipid-based nanoparticles are among the most attractive drug carriers in the field of dermal and transdermal drug delivery [47,48]. This is in compliance with their structural similarity to skin barriers and compatibility with the majority of dermal bases. The nanotechnologies in question are based on the physico-chemical interaction between liquid, soft or hard lipids (phospholipids, mono, di or triglycerides, fatty acids or alcohols, waxes, cholesterol) and surfactants, in the presence or not of other excipients under different type of processing [49]. Depending on the nature of the lipids, the experimental conditions, and the ingredients ratio, nanosized aggregates may occur with different morphology, from liquid core-elastic wall vesicles (liposomes, niosomes, ethosomes) to thermodynamically stable liquid-in-liquid systems (nanoemulsions) or variously structured solid or soft particles (solid lipid nanoparticles or other types of nanostructured lipid carriers). However, all of the above-mentioned lipid-based nanocarriers possess some universal features, such as the ability to modify drug release, encapsulate efficiently hydrophobic molecules (and some hydrophilic ones, as well), improve drug solubility and permeation, and increase drug stability by providing a protective microenvironment [47,48,49]. As the lipid-based nanotechnologies often involve steps in preparation at higher temperatures and/or sonication [50], the chemical stability of the active compounds under such conditions should be investigated and considered. This is highly relevant, although not widely discussed for the phenolic compounds and their introduction to lipid-based nanostructures.

## 2. Phenolic Compounds

The term ‘phenolics’ relates to all biologically active compounds having at least one phenolic ring in their structure. Being a major class of secondary metabolites with a vital role in growth regulation, defense, and signaling, they are widely distributed among the plant kingdom [51,52]. Phenolic compounds originate from the shikimic and acetic acid biosynthetic pathways. Besides being united by a common genesis, and, therefore, elements in the structure [53], the representatives of this group share similarities in their pharmacological activity, mechanism of action, and therapeutic effects. An essential quality of phenolics is the reduction of oxidative stress in vivo [4,5] by scavenging reactive oxygen and nitrogen species (ROS, RNS) and free radicals, inhibition of key enzymes (xanthine oxidase, lipoxygenases, cyclooxygenases, monoamine oxidase, nicotinamide adenine dinucleotide phosphate oxidase and other), suppressing ROS/RNS generation, activating natural antioxidant systems (as superoxide dismutase, catalase, glutathione peroxidase [13,54]) and chelating metal ions (well-known to act as catalysts of oxidation processes [55]) [13,56,57]. The antioxidant ability of phenolics is determined, in the utmost, by the presence of electron-donating phenolic group/s, whereas the electron-donation process is highly dependent on the electron-density distribution in the aromatic core and thus the nature of the other substitutes in the structure [58,59]. Phenolic compounds are known to build stable radicals after neutralizing reactive species and free radicals, and terminate the oxidative chain reactions by interaction with one another [60]. Since oxidative stress is a fundamental element in the genesis of inflammatory, allergic, oncogenic, and atherogenic pathologies [61,62,63,64], the emphatic antioxidant properties of phenolics are the underlying prerequisite for their numerous health benefits in humans. It is known that many molecular mechanisms other than antioxidant activity are involved and contribute to the anticancer, anti-inflammatory, antiallergic, immunomodulatory, antimicrobial, antiaging/regenerative, antiatherogenic, and vasoprotective potency of phenolics. However, they are particularly related to the individual structures, the presence, and the type of glycoside parts, which we discuss below by groups and members. More importantly, the mechanisms of action of phenolics suit the functional and structural deficiencies related to many skin diseases and conditions, wherefore they are widely investigated and applied in the field of dermatology.

## 3. Fields of Application of Phenolic Compounds in Dermatology

The prevalence of skin diseases has substantially increased in recent years (by almost 50% for the past three decades) [65,66]. Today, they represent the fourth most common cause of all human diseases and affect approximately one-third of the world population [67]. The need exists for new therapeutic alternatives to be sought, as the treatment of the most frequently encountered skin disorders often includes the local or systemic use of steroids and antibiotics, both known to exhibit explicit side effects and long-term health risks [68,69,70,71].

The majority of most common dermatological diseases are associated with oxidative stress (ROS/RNS generation) and activation of the immune-inflammatory cascade [61,62,72]; such diseases are referred as inflammatory skin diseases [73,74]. These include atopic dermatitis (eczema), acne vulgaris, psoriasis, allergic contact dermatitis, urticaria (hives), seborrheic dermatitis, lupus erythematosus [75,76], alopecia areata [77], rosacea [78], vitiligo [79], skin malignancies (for whose pathogenesis inflammation is a key mechanism) [80,81] and others. In this regard, the phenolics’ antioxidant activity makes them suitable therapeutic agents for the local treatment of these pathologies. Furthermore, reduction of oxidative stress in the skin tissues is also important in the name of prevention against UV-radiation-mediated aging, loss of natural antioxidant capacity, DNA damage, and initialization of carcinogenesis. The most promising protective agencies, in this regard, are representatives of the anthocyanins and catechins (flavan-3-ols) [82,83], which, indeed, are among the strongest antioxidants in the flavonoid class [58]. Other molecular mechanisms of action, unrelated or indirectly related to antioxidant activity, are also established for the set of representatives, and they extend further the phytophenolics’ therapeutic field. The most important of such effects, with relevance to skin diseases and dermal drug delivery, are described below.

### 3.1. Interaction with Bacterial Cell Walls, Cell Membranes, and Synergism with Antibiotics

The ability of some phenolic compounds to interact with bacterial cell walls and cell membranes is fundamental to their antibacterial activity [84]. In several studies, phenolic compounds (epigallocatechin gallate, epicatechin gallate, gallic and caffeic acids) have been demonstrated to interfere with bacterial cell wall integrity, causing damage in its structure and leakage of cellular constituents [85,86,87]. The interaction is attributed to a bonding of the active phenolic molecules with the peptidoglycan layer through hydrogen and/or covalent bonds (for Gram-positive bacteria) and/or the lipopolysaccharides (for Gram-negative bacteria) [84,85]. Furthermore, inhibitory actions on the penicillinase enzyme and the efflux pump are found to contribute to a decrease in antibiotic resistance and synergistic antibacterial activity of phenolics with antibiotics [88,89,90,91]. Much more of the phenolic group representatives are proven to owe their antibacterial potency to alteration of bacterial cell membrane permeability, fluidity, ion transport, and respiration [82]. Rigidification or fluidization may be observed depending on the chemical structure of the phenolic molecule (polarity, molecular mass, and conformation) and its positioning among the lipid bilayer [92,93]. For example, the flavonoids kaempferol, chrysin, quercetin, baicalein, luteolin, epigallocatechin gallate, gallocatechin, theaflavin, and theaflavin gallate, when in contact with the bacterial cell membrane, decrease its fluidity, while the isoflavonoids puerarin, ononin, daidzein, genistein, and the stilbene resveratrol have shown the opposite effect [90,91]. Destabilitization of the bacterial cell membranes could also result from phenolics stepping into reaction with enzymes responsible for cell membrane stability and integrity [68,94]. In addition, some phenolic acids (caffeic and gallic acids) acidify the bacterial membrane, leading to its disruption and changes in permeability and ion transport [95]. Membrane damage and subsequent potassium loss from the bacterial intercellular space are also reported for galangin [96], a flavonoid (flavanol) found in propolis, to which the antibacterial properties of the latter could be partially attributed [97].

### 3.2. Interaction with Microbial DNA/RNA Polymerases and Topoisomerases, Proteases, Transcriptases, Surface Proteins (Adhesins), and Other Virulence Factors

In general, the biologically active aglycons of phenolic compounds possess a structure rich in reactive functional groups, multiple phenolic groups, carbonyl groups (e.g., xanthones, anthraquinones, most flavonoids), free or esterified carboxylic groups (phenolic acids), among others. They easily step into hydrogen bonding with other biomolecules (nucleotides, proteins, including adhesins and receptors, enzymes as DNA/RNA polymerases and topoisomerases, transcriptases, proteases, and many others) [94,98,99] or complexation with metal ions (iron ions) [100] that are essential for the infectious cycle of pathogenic bacteria and viruses (adhesion, entry, replication and spread) [84,101]. This is a wide-ranging and nonspecific complex of potential interactions of phenolics that has led many researchers to understand their antiviral and antibacterial properties. Examples relevant to skin infections to support this theory include curcumin (diferuloylmethane), which exerts its antiviral activity against human herpesvirus -1 (and other DNA viruses) by blocking the histone-acetyltransferase activity of specific transcriptional coactivator proteins (p300 and the CREB-binding proteins) [102,103]. Curcumin, again, is also found to inhibit the adhesins-mediated adsorption and replication of human herpesvirus 1 and 2 [104,105]. Epigallocatechin gallate, which was previously mentioned to possess a destructive effect on bacterial cell walls, exhibits its antibacterial action against methicillin-resistant Staphylococcus aureus also by inhibiting multiple staphylococcal virulence factors [6,90]. Quercetin, kaempferol and other flavonoids inhibit staphylococcal topoisomerases [106,107,108].

Today, the antibacterial, antiviral, and antifungal activity of phenolic compounds is considered a fact after being a subject of study for decades [109,110]. They have shown activity against the most frequent causative agents of skin infections, such as bacteria of the genera Staphylococcus, Pseudomonas, Enterococcus, the Herpes virus 1 and 2, the dermatophytes genera Trichophyton, Epidermophyton, and Microsporum [2,95,111]. Therefore, their topical use is highly beneficial for the purposes of infectious skin diseases’ healing (dermatophytosis, impetigo, herpes infections, infected wounds, and others). Special attention is dedicated to dermal products containing phenolic compounds in cases of antibiotic-resistant infections, which have become more and more commonly encountered problem [2,112]. However, the exact mechanism of antimicrobial activity of a given phenolic compound is not always thoroughly investigated and fully understood. Even so, the significance of many other phenolic representatives as antimicrobial agents, beyond the list of examples given above, needs to be acknowledged. Among them are the main active compounds in Hypericum perfuratum preparations: hypericin, pseudohypericin and hyperforin [113,114,115,116], resveratrol [117,118], vitexin and isovitexin [119], hesperidin [120,121], and eugenol [122].

### 3.3. Effects on Skin Renewal, Proliferation, Collagen, and Elastin Synthesis

Indisputably, the regenerative properties of the phenolic compounds are among their strengths and justify the role of this phytochemical group in the therapy of wounds, incised or chronic, burns, infected wounds, etc. (for which, of course, the antimicrobial properties also contribute) [123,124,125,126]. Skin regeneration is a complex process that involves a vascular response (hemostasis and coagulation), cellular response (inflammation), proliferation phase (re-epithelialization), neovascularization (angiogenesis), granulation tissue formation, and remodeling (strengthening by conversion of collagen type III to type I) [127]. The reduction of oxidative stress in the early stages of injury may facilitate physiological responses (swelling, redness, pain) because of the direct relationship of reactive species and free radicals with the inflammatory mediators’ secretion [128] (vasoactive amines and proteins, cytokines, prostaglandins [128,129]). The late phases of wound healing are based primarily on the proliferation and migration of fibroblasts, keratinocytes, and endothelial cells, and the activation of collagen and fibronectin synthesis [130]. The signaling pathways responsible for these processes also include cytokines and growth factors release from epithelial and nonepithelial cells, and are dependent on oxidative balance and supported by antioxidant-acting molecules. It is clear now that many phenolic antioxidants favor skin regeneration and renewal by reducing inflammation, inhibiting matrix metalloproteases, collagenases, elastases, increasing the expression of endothelial growth factor and the transforming growth factor, and thereby promote re-epithelization, angiogenesis, maturation, and thus tissue regeneration. Such activity is also highly desirable in the fight against age-related changes of the skin [131] (wrinkles appearance, loss of elasticity, thinning). Examples of phenolic compounds or herbal preparations that have been demonstrated to exert these mechanisms in vitro and/or in vivo are luteolin [132], epigallocatechin gallate and extracts rich in it, and other tannins [133,134], crude grape pomace and its main constituent gallic acid [135], lignans in seedcake extract [126], other phenolic-rich content extracts from the cacao pod [136], Phyllanthus emblica, Manilkara zapota [137], Clausena excavate [138], Sphaeranthus amaranthoides [139], Meum athamanticum, Centella asiatica, Aegopodium podagraria [140] and many more. Despite the undeniable role of the antioxidant properties of phenolics for skin regeneration, other supplementary mechanisms are found to be involved in the healing/protective processes. For instance, several genes involved in skin renewal (Kruppel-like factor 10, E2F-4 transcription factor, and epidermal growth response factor) have been up-regulated in human dermal fibroblast cell cultures when treated with Populous nigra preparations (rich in caffeic, p-coumaric, cinnamic, isoferulic acids, pinocembrin, salicin, and other phenolic compounds) [141]. Similar modulatory effects on gene transcription have been established for ellagitannins from oak wood, caffeoyl- derivatives from mate leaf, and phenolic acids from benzoin resin [142].

### 3.4. Effects on Melanin Synthesis

Melanin is a term referring to a complex of natural pigments with a crucial role in skin coloring and photoprotection. It is deposed in the keratinocytes after migration from the melanocytes cells, where it is produced from tyrosine through multiple oxidation reactions catalyzed by the enzyme tyrosinase [143,144,145]. Many phenolic compounds have shown competitive inhibitory activity on tyrosinase due to a structural resemblance with its initial substrate tyrosine, and chelation of the copper ions present at the binding sites of the enzyme [10,146,147]. In this regard, phenolics have found their application as tyrosinase inhibitors in the treatment of hyperpigmentation skin disorders [10]. Furthermore, melanogenesis suppression is considered to be one of several mechanisms of the phenolics’ anticancer activity in the therapy of melanoma-type skin tumors [2,148,149]. Among the strongest tyrosinase inhibitors from the phytophenolic group are isoliquiritigenin [150] (chalcone structure), galangin [151], kaempferol [152], luteolin [153], apigenin [153], resveratrol [154], isoeugenol [155], p-coumaric, caffeic and rosmarinic acid [156,157]. With respect to antimelanogenic activity, glycoside forms of some phenolic compounds have shown higher efficacy due to increased tyrosinase inhibitory capacity [158,159], and/or lower toxicity [160]. It should be noted that significant cytotoxicity on melanocytes and risk of leucodermia (induced vitiligo), ochronosis (diffuse skin bluish-black discoloration), and carcinogenesis are inherent for many skin-lightening substances, including those of the natural phenolics class [145,161,162]. The very potent whitening agent hydroquinone, for instance, has fallen into the list of forbidden substances in cosmetics as a result of confirmed relation between its topical use and the above-mentioned adverse reactions [160,163]. In general, the contemporary research in this area is focused on seeking synthetic or semisynthetic phenolic molecules that will inherit the natural compounds’ high depigmentation activity with less toxicity and higher stability [10,164].

Paradoxically, in some cases, the application of phenolic compounds has shown beneficial effects in the therapy of vitiligo [165], an autoimmune-determined disturbance in melanogenesis manifesting itself as white patches on the skin [166]. Such cases occur when (1) phenolics are used as whitening agents in order simulate merging of the white vitiligo spots and lead to total whitening (depigmentation therapy; synthetic or semisynthetic phenolics), or (2) antioxidants protect the melanocytes and keratinocytes [167] (mostly natural or semisynthetic phenolics). Oxidative stress is considered one of the main inducers of auto-reactive T-cells against the epidermal melanocytes and the destruction of the latter [166]. Therefore, in terms of an ongoing oxidative stress-related autoimmune response against the melanocytes, phenolic compounds may exhibit a protective action toward melanogenesis [166,168] (e.g., curcumin and its metabolite tetrahydrocurcumin [169], quercetin [170,171], the green tea polyphenols, epicatechin, epicatechin-3-gallate, and epigallocatechin [170]).

### 3.5. Photosensitization

Photosensitization may occur due to a phototoxic reaction (an acute light-induced tissue response to a photoreactive chemical) or photoallergy (an immunologically mediated response to a chemical, initiated by the formation of photoproducts following a photochemical reaction). It is a concern for compounds that possess high molar absorptivity (>1000 L mol^−1^·cm^−1^) at each wavelength within the range of natural sunlight (from 290 to 700 nm), generate reactive species after absorption of light, and distribute/accumulate in the skin [172]. Such properties among the phytophenolic group are characteristic for anthracene derivatives [173] (anthraquinones—aloin A, aloe-emodin, hypericin), lignans (in the composition of silymarin), and curcumin, and some of its derivatives [174]. They may cause photosensitization (sunburn-like symptomatic such as skin irritation, erythema, pruritis, edema [175]) after systemic, as well as dermal administration [172].

### 3.6. Antitumor Activity of Phenolics

Skin cancers, being the most serious group of skin diseases (incl. basal cell carcinoma, squamous cell carcinoma, malignant melanoma) [2], are among the most tempting research areas for scientists to explore the potency of alternative treatment options, and the therapeutic application of a promising group such as phenolic compounds makes no exception. The anticancer activity of phenolics is primarily due to their antioxidant properties and high reactivity (hydrogen and/or covalent bonding with essential biomolecules), whereas one or both of which lead to additional mechanisms determining their complex action and high efficiency. In particular, phenolic compounds are proven to interfere with the cancer cell life cycle by inducing caspases activity and apoptosis of cancer cells (curcumin [176,177,178], luteolin [179], vitexin [180], epicatechin gallate [177,181], gallic acid [182], eugenol [183]) and regulation of gene expression in cancer cells (for example, *eugenol* is found to induce down-regulation of c-Myc, H-ras and Bcl2 expression and up-regulation of p53); to inhibit epidermal growth factor-induced neoplastic transformations in cell lines (caffeic acid [184]); to inhibit tyrosinase and melanogenesis (a mechanism relevant to melanoma type of skin cancer; examples are given in a previous section); to inhibit the proteasome; an enzyme complex responsible for the degradation of essential proteins involved in cell development, and lead to subsequent suppression of cancer cell growth and spread (catechin-3-gallate and epigallocatechin gallate [185], gallic acid [186], apigenin [187], quercetin [187], curcumin [188]), and to destabilize lysosomal membrane through permeabilization and cause cancer cell death (pterostilbene, a dimethoxylated analog of resveratrol [189]). These examples are only a few concrete representatives chosen for subjects of specific investigation, whereas the whole complex of proposed mechanisms of action is potentially valid for a much larger sample of natural and modified phenolic compounds.

### 3.7. Phenolics as Pro-Oxidants

Amongst the abundance of scientific reports regarding the mechanisms of action of phenolic compounds (including those mentioned in this review), it is not hard to follow an apparent controversy. For example, some phenolic compounds are found to promote injured skin regeneration by inducing the epidermal growth factor and transforming growth factor, whereas the same or similar compounds are shown in different studies to suppress epithelial cancer cell development and spread due to opposing effects on growth regulation factors [190,191]. There is a theory based on the concept of a switch between anti-and pro-oxidant properties of phenolics as a function of microenvironmental factors. A decrease in antioxidant properties and switch to pro-oxidant activity of phenolic compounds is observed under conditions of decreased pH (intrinsic for cancer cell lines) and upon complexation in the presence of transition metals (Cu, Fe: Cu^2+^→Cu^+^, Fe^3+^→Fe^2+^; extracted from the herbal drugs, for example), which indeed stabilizes the phenoxyl radicals and enhances the production of reactive species [192]. Formation of metal-phenolic networks is more likely for 3-hydroxy-, 4-carbonyl flavonoids (flavonols—e.g., quercetin, kaempferol, galangin, morin, myricetin) [193]. The environment-determined switch to pro-oxidant properties is a matter of potential toxicity and is among the possible explanations for anticancer activity [194].

## 4. Dermal Drug Delivery of Phenolic Compounds

Despite the countless proofs for the multidirectional therapeutic potential of phenolic compounds in dermatology, a few simple facts must be acknowledged. (1) In order for them to exert their molecular mechanisms on targeted structures, they must reach the latter and accumulate in sufficient concentrations. (2) They must possess sufficient stability during storage and until deposition in the relevant skin layer, and hence be included in proper dosage forms by suitable technological operations with the aid or not of drug-delivery vehicles. (3) Additional factors, such as potential toxicity under certain conditions, should be considered.

### 4.1. Biopharmaceutical Considerations of the Dermal Drug Delivery

Absorption is not a primary physiological function of the skin; on the contrary, the epidermal layer, in particular, is an effective barrier for the intrusion of foreign matter (including potentially hazardous matter). Therefore, dermal drug delivery is challenging and sets numerous requirements for the chosen therapeutic agents and dermal bases/vehicles. The skin possesses a complex structure of multiple layers with different morphology and function, starting with the corneum, the outermost nonviable, keratinized epidermal stratum responsible for the limited permeability of the epidermis. Molecules can pervade in it either by paracellular transport (through the lipid matrix; preferable route for mostly lipophilic compounds, log P ≥ 2) or via the transcellular route (through the corneocytes, the constructive type of cells in stratum corneum, often compared with bricks walled up in the “mortar” of lipid milieu; alternative transportation for more hydrophilic molecules). At this stage of entry (referred as penetration), it is evident that lipophilic properties of the applied therapeutic agent are preferable. However, further transportation of the substrates to the viable epidermis and the derma (permeation), and/or their percutaneous absorption, requires sufficient water solubility (~0.5–1.0 mg/mL) otherwise, they are retained in the congenial surrounding of stratum corneum and not be able to overcome the amphiphilic nature of the underlying cutaneous stratums. Other possible, but rather supplementary mechanisms of drug permeation through the skin, are the transfollicular transport or passage across the sweat glands [195,196]. The pathophysiology of the most common skin diseases (impartially reviewed in the previous sections) suggests that the therapeutic targets for phenolic compounds are settled either in the viable epidermis or the derma, rarely in the hypodermis (e.g., keratinocytes, melanocytes, immune cells, mast cells, endothelium, hair follicles, etc.). Therefore, permeation is essential for a practical manifestation of their activity. Besides, a balanced hydrophilic-lipophilic profile (and a respective suitable partition coefficient, ideally in the range of log P 2–3 [197,198]) is only one of the desired qualities for successful skin permeation. Further limitations are set by the molecular weight (<500 Da, but often a lower limit is set with respect to the other molecular particularities of the active compound) and the potential for ionization [199].

### 4.2. Physico-Chemical Properties of Some Common Phenolic Compounds and Their Glycosides

The separate classes of phenolic compounds differ substantially in their physico-chemical properties and skin permeation. The simple phenols, including phenolic acids, for example, are characterized by lower molecular weight and higher water solubility compared to the majority of other phenolics [200] (Table 1). A limitation for their cutaneous permeation is the presence of multiple ionizable groups (alcohol and carboxyl groups). The flavonoid aglycons (e.g., quercetin, kaemferol, luteolin, apigenin) are distinguished with extreme hydrophobicity, with the exception of the class of catechins that cross the water solubility barrier of >1 mg/mL (needed for effective skin permeation). The same undesirable practical insolubility in water is also inherent for the majority of other polyphenols (xanthones, anthraquinones, stilbenes, lignans, tannins, phloroglucinols). Glycosylation, as a biosynthetically occurring metabolic process, leads to the formation of more hydrophilic derivatives. However, the water solubility improvement of the phenolics’ glycoside forms is sometimes insufficient (e.g., apigenin→vitexin, hesperetin→hesperidin, Table 1). In general, many approaches involving chemical modification of the phenolic compounds (sulfonation, phosphorylation, complexation, incl. with metal ions, biomacromolecules or cyclodextrins [99,201,202,203]) are studied for their potential to obtain analogs or prodrugs with increased water solubility and bioavailability. On the other hand, the “blocking” of reactive groups by etherification, esterification, and other processes, is a well-known approach to improve skin permeation due to reduced ionizability [204], whereas such operations, depending on the substrates’ nature, may lead to an increase or a decrease in water solubility [205]. Examples could be given for caffeic acid and chlorogenic acid, where the latter, being an ester of the former with quinic acid, despite its higher molecular mass has better skin permeation [206]. A few methoxylated quercetin derivatives were shown to possess increased skin permeation compared to the native quercetin by Lin et al. [40]. The same authors reported even better dermal penetration of rutin (quercetin-3-*O*-rutinoside; Mw 610.52) compared to quercetin (Mw 302.24), due to higher hydrophilicity, although these results contradict other research findings [207].

### 4.3. Stability of Phenolics

The chemical stability of phytophenolics is a priority concern since these highly reactive molecules take part in all types of degradation processes (incl. oxidation/autooxidation, hydrolysis, isomerization) and lose their therapeutical efficacy over time [34,259,260,261]. Furthermore, for the majority of chemically sensitive phenolics, light irradiation has been identified as a determining factor for decomposition process mechanisms and rates [259,262]. Therefore, many phenolic compounds are known to be susceptible to photodegradation (resveratrol [263], curcumin [264], hypericin [255], hyperforin [31], eugenol [265], quercetin [266], and many others) [267]. Furthermore, polymerization is another undesirable event for some simple phenols and polyphenols (catalyzed or not by oxidation processes) [259,268], which leads to substantial changes in their pharmacological activity, molecular mass, and skin permeation potential.

Considering this information, the choice of a dermal drug delivery vehicle (viz. the inclusion of permeation enhancers in the composition or the utilization of nanoparticulate delivery systems, the type of solvents, etc.) determines the penetration potential and stability of the chosen phenolic compound(s). Among the numerous investigated approaches with respect to improved skin permeation and stability (including chemical modification, prodrug development, complexation, solvent type optimization, inclusion of the therapeutic substrates in micro and nanosized carriers), the application of lipid-based nanotechnologies for the dermal delivery of phenolics has gained the most interest and practical significance. In favor of the lipid nanoparticulate systems are the ability to incorporate and stabilize sensitive molecules, “disguise” some of their unfavorable structural particularities for dermal transport, and the opportunity they provide for modified drug release. Another interesting aspect of dermal drug delivery of phytochemicals with a phenolic structure via lipid-based nanotechnologies is hidden in the fact that phenolic antioxidants inhibit lipid peroxidation in the corpus of these nanovehicles and provide longer endurance of the latter. Therefore, it can be stated that the relation between phenolic phytochemicals and lipid nanocarriers could, under some circumstances, be described as symbiotic.

## 5. Lipid-Based Nanotechnologies

Lipid-based nanosystems are the subject of great interest in dermal and transdermal drug delivery, as they provide a successful approach to overcome the limitations of conventional topical formulations, improving at the same time the characteristics of the loaded cargo (drugs and biologically active compounds), its skin permeation and consequently therapy efficacy [49]. The lipid nature of nanoscale drug delivery systems, such as solid lipid nanoparticles (SLNs), nanostructured lipid carriers (NLCs), liposomes, or nanoemulsions, ensures their excellent skin tolerability, biodegradability and, if necessary, allows their easily structural modification/optimization in the formulation process due to the great variety of lipid constituents. Furthermore, their salient characteristics, such as improved solubility, stability, and bioavailability of the incorporated active ingredients, as well the achieved controlled release profile, would be particularly beneficial for the inclusion of phytochemicals with phenolic structures, allowing them to fully deploy their favorable dermal effects [269]. In this regard, a summary of the specifics of the most commonly used lipid-based nanosystems, and their application as nanocarriers for encapsulation of phenolic compounds in dermal/transdermal delivery, is provided below.

### 5.1. Liposomes

**Figure 1:**
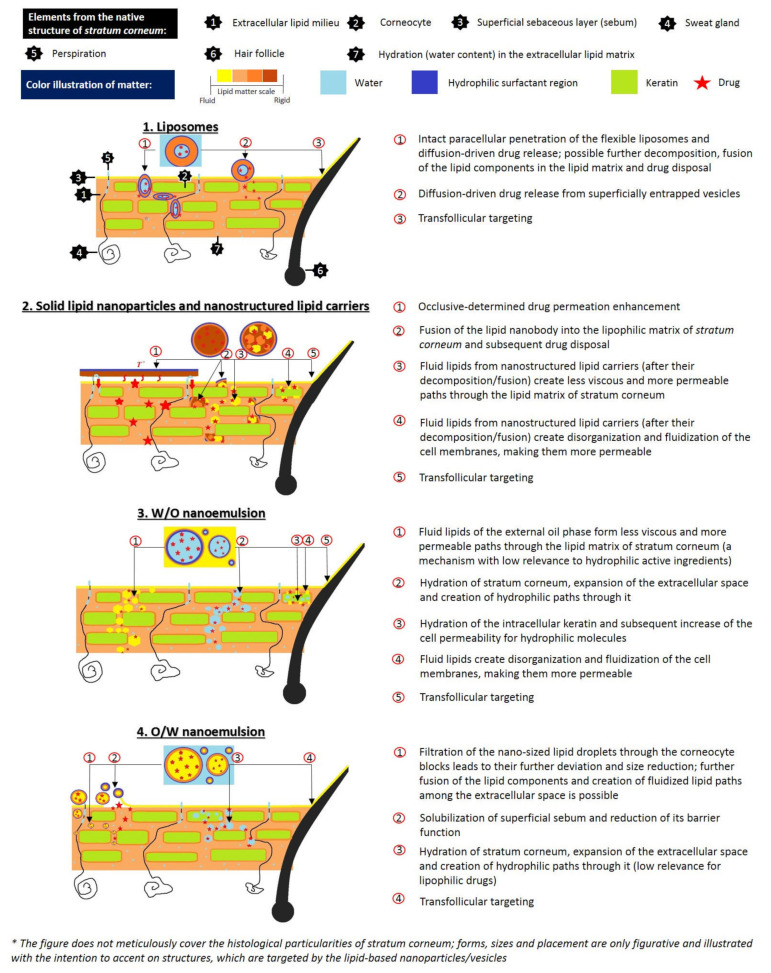
Possible mechanisms of drug permeation enhancement through *stratum corneum* by lipid-based nanoparticles/vesicles.

Liposomes may be considered among the first lipid-based nanosystems. After their discovery in the 1960s by Bangham, they were initially proposed as a model for biological membranes due to their compositional similarity. However, later in the 1970s, thanks to their excellent biocompatibility properties and entrapment ability, they were studied as potential drug delivery platforms [270,271]. Structurally, liposomes are spherical vesicles consisting of one or more phospholipid bilayers surrounding an inner aqueous compartment [272,273]. The vesicular structure, resulting from the amphipathic properties of the bilayer forming lipids, provides the opportunity to encapsulate hydrophobic and hydrophilic molecules [274]. The origin of the phospholipids (from various natural or synthetic sources) and their chemical structure influence liposomal properties and membrane fluidity. In their study, Jacquot et al. [275] investigated the effect of marine (salmon) and plant (rapeseed) isolated phospholipids on membrane fluidity and the mechanical properties of liposomal bilayers compared to dioleylphosphatidylcholine and dipalmitoylphosphatidylcholine-based membranes as references. The authors reported that the membrane fluidity was influenced by the saturation of the fatty acid chains; the highest values were obtained in the membranes based on dioleylphosphatidylcholine (unsaturated acyl chains) and lowest in the bilayers formed from dipalmitoylphosphatidylcholine (saturated acyl chains). Regarding mechanical properties, phase segregation was reported for the rapeseed membranes, whereas the unsaturated salmon bilayer was characterized by a homogenous structure. The rigidity of the liposomal bilayer may be further increased by the inclusion of cholesterol, which can fill the cavities resulting from the loose packing of phospholipids, thus improving liposomal in vitro and in vivo stability [276,277]. Liposomal physicochemical parameters (size, surface charge), membrane characteristics, and interaction between the encapsulated active agent and liposomal constituents, influence the mechanism and extent of the drug delivery process [278,279]. The appropriate size of liposomes for topical application is below 300 nm to reach deeper skin layers. However, vesicles with a size below 70 nm are characterized with maximum deposition in the epidermis as well the dermis [280]. Several mechanisms are proposed to explain the active agent transfer from liposomes to the skin, such as vesicle fusion with lipids of stratum corneum as a result of their similar structure; a fluidizing effect, leading to impaired skin integrity; intact liposomal penetration into different dermal layers (associated with their flexibility; possible alterations in size and structure); improved drug delivery through hair follicles or sweat ducts facilitated by liposomal vesicular structure, and free active agent penetration after its release from liposomes (Figure 1) [271,281,282]. To evaluate the influence of zeta potential on transdermal delivery, Park et al. [283] investigated *resveratrol* permeation from conventional liposomes as well from vesicles coated with chitosan. According to the authors, higher resveratrol skin deposition was estimated from the chitosan-coated vesicles due to the repulsive electrostatic interaction between cationic chitosan vesicles and negatively charged epidermal lipids. The incorporation of phenolic compounds in liposomes, as well their interaction with phospholipids, has been studied by many researchers. In their study Malekar et al. [274] investigated the localization of five chemically diverse phenolic compounds (raloxifene, garcinol, quercetin, trans-resveratrol, bisphenol A) in dipalmitoylphosphatidylcholine-based liposomal bilayer and their influence on colloidal stability. As reported by the authors, the phenolic compounds, localized in the central regions of the bilayer (resveratrol and quercetin), negatively influenced liposomal colloidal stability due to decreased contact with phosphate head groups. Inversely, phytochemicals in the glycerol region of the acyl chains (raloxifene, garcinol, bisphenol A) contributed to better stability thanks to the enhanced exposure with phosphate head groups or electrostatic repulsion forces. Phan et al. [92] studied the interaction mechanism of two different classes of polyphenols (flavonoids and trans-stilbenes) with liposomal membranes. The flavonoids’ gallate, galloyl, and hydroxyl groups are connected via hydrogen bonds with membrane lipids, leading to compact phospholipid assembly, reduced surface area, and forming a stiffer bilayer. The benzyl open ring structure of trans stilbenes, on the other site, determines its deeper intercalation into the hydrophobic bilayer, causing extension of the membrane area and enhancing its fluidity.

### 5.2. Solid Lipid Nanoparticles

Solid lipid nanoparticles, reported for the first time in the 1990s by Professor R.H. Müller and Professor M. Gasco, were proposed as an alternative approach to overcome the limitations associated with liposomes (e.g., phospholipid oxidation, costly materials, and production process, limited physical stability, difficulties in process scale-up) and polymeric nanoparticles (polymer toxic degradation process) [284,285]. As suggested by their name, SLNs are composed of individual or a mixture of lipids, solid at ambient and body temperature, dispersed in water or an aqueous phase containing surfactant [286]. Most commonly used lipids for SLNs preparation include triglycerides (trimyristin, tristearin), fatty acids (stearic, palmitic acid), waxes (beeswax, cetyl palmitate), and mono/di/triglycerides mixtures (glyceryl behenate—Compritol 888 ATO, glyceryl palmitostearate—Precirol ATO 5) [287]. Solid lipid nanoparticles can be suitable carriers for both hydrophobic and hydrophilic compounds. According to their structure and drug localization they can be classified as homogenous matrix models and core-shell models (drug-enriched shell and drug-enriched core) [288]. In the first case, the active agent is molecularly dispersed within the matrix or present as amorphous clusters. Homogenous matrix SLNs are obtained by cold or hot (when encapsulating highly lipophilic molecules) homogenization methods. In the second model, an outer shell containing the active agent surrounds a lipid core. The specific morphology of drug-enriched shell nanoparticles results from phase separation during the cooling phase; initially, the lipid in the center precipitates, shaping the inner, compound-free compartment. However, at the same time, drug concentration in the residual melted lipid increases and, after solidification, a drug-enriched shell is formed. In the third model SLNs, due to drug super-saturation in the lipid melt, a crystallization of the active agent is observed before the crystallization of the lipids, resulting in a drug-enriched core enclosed by a lipid (drug-free) shell [288,289,290]. The type of SLNs and their composition and physicochemical parameters affect their skin permeation. Due to the lipid nature of SLNs, as possible mechanisms of skin penetration (analogous to liposomes) the fusion of nanoparticles with lipids of stratum corneum, the lipid fluidizing properties of lipids, and transfollicular transfer are proposed. However, as a specific penetration mechanism, characteristic of SLNs may indicate their occlusive effect. Thanks to their large surface area and nanosized dimensions, they possess occlusive characteristics leading to improved skin hydration and enhanced penetration into dermal layers (Figure 1) [289,291]. In their study Kakkar et al. [292] used tetrahydrocurcumin-loaded SLNs characterized by sufficient occlusivity and anti-inflammatory effects. Their further incorporation in hydrogel formulation led to seventeen times higher skin permeation ability compared to plain tetrahydrocurcumin gel. Regarding their structure, suitable features for topical application include the drug-enriched shell nanoparticles, providing rapid drug release, which along with the occlusive effect is a favorable characteristic when increased drug penetration is necessary [288,293]. The disadvantages of SLNs, such as the tendency for drug expulsion or low drug loading capacity due to the ideal crystalline structure of the lipids, provide possibilities for further development, including the development of second-generation nanoparticles and structured lipid carriers (NLCs), which overcome the limitations mentioned above [284,294].

### 5.3. Nanostructured Lipid Carriers

Nanostructured lipid carriers are composed of solid and liquid lipids dispersed in the aqueous phase, stabilized by surfactants [295]. The inclusion of a liquid lipid in their composition disrupts the highly ordered crystalline structure characteristic of the SLNs. It leads to a less organized lipid matrix, providing space for more drug accumulation [296,297]. NLCs may be categorized into three types: imperfect crystal, amorphous and multiple types, by the lipid blending ratio and their method of preparation. Type I NLCs are prepared from lipids (predominantly solid, with small amount of oil phase), differing in their structure concerning chain length or saturation, leading to the formation of a disordered “imperfect” lipid matrix characterized by high encapsulation capacity. An amorphous structure, indicative of the second type of NLCs, is obtained by including specific lipids to the composition so the crystallization after cooling can be prevented. Consequently, leakage of the active agent also is minimized. The multiple model NLCs contain a significant amount of liquid oil phase in their composition, which facilitates a phase separation during the formulation process leading to nanosized liquid oil compartments among the solid lipid matrix. The solid matrix may be referred to as a barrier, providing a controlled release of the active agent, whereas the liquid lipids ensure better solubility of included lipophilic molecules and therefore determine higher drug encapsulation [284,293,298,299]. The effects of the included liquid lipid in the NLCs composition, as well as the selected oil phase on the encapsulation efficiency and antioxidant activity of the phenolic compound sesamol, was investigated by Puglia et al. [300]. The authors developed two different NLC formulations composed of the same solid lipid (Compritol^®^888 ATO) with varying liquid phases (Miglyol 812 or sesame oil), as well one model Compritol^®^888 ATO-based SLNs for reference. Sesamol encapsulation was higher in the NLCs formulations than the SLNs, with the highest entrapment efficiency values (>90%) and improved antioxidant activity for the formulation containing sesame oil as the liquid oil phase. The observed results may be attributed to the structural similarity between the active agent and selected oil phase, which determines the potent synergic effect. NLC composition also influences the occlusive effect and their dermal/transdermal delivery. In their study, Loo et al. [301] investigated the influence of lipid concentration (20% and 30%), solid lipid/oil ratio, and additives (lecithin, propylene glycol) on skin hydration and transepidermal water loss. According to the authors, NLCs with higher lipid content (30%), high solid lipid concentration (90%), and additives (slightly favorable outcomes in case of lecithin) were characterized with good occlusive properties and led to improved skin hydration and reduction of transepidermal water loss during the seven-day study period. Regarding particle size, another important factor determining the extent of skin permeation, both NLCs and SLNs may be prepared in the appropriate form for dermal application range (100–500 nm) via a high-pressure homogenization (hot and cold) method, which is also suitable for large scale production [289,298]. There is some controversial literature regarding the possibility of encapsulating temperature-sensitive compounds (such as some phenolic compounds) via the hot method. However, this technique is considered applicable due to the short heating time, except for highly temperature-sensitive, hydrophilic molecules, which might migrate to the aqueous phase during homogenization [288]. The mechanisms by which NLCs improve drug permeation through the skin are similar to those, discussed for SLNs (Figure 1).

### 5.4. Nanoemulsions

Nanoemulsions are isotropic colloidal dispersions composed of water and oil stabilized using surfactant/cosurfactant. One of the liquids is dispersed into nanosized droplets, usually between 20 and 200 nm [302]. The tiny droplet size causes their transparent/translucent appearance (analogical to microemulsions). However, differences between these two systems may result from the surfactant concentrations used (ca. 20% in microemulsions, vs. 3–10% in nanoemulsions), as well their dissimilar thermodynamic stability (nanoemulsions are thermodynamically unstable, while microemulsions are thermodynamically stable) [303,304]. Nanoemulsions are an attractive drug delivery system for topical application due to their miniature droplet size, homogenous size distribution, and large surface area, which ensure their uniform spreading onto the skin surface that facilitates drug penetration [305]. Similar to the other lipid-based nanosystems, an inverse relationship between the size of carriers and their transdermal penetration has been reported. According to Su et al. [306], who investigated transport through the skin of nanoemulsions loaded with environment-responsive and fluorescent dyes, formulations with droplets size of 80 nm can diffuse (but not penetrate) into the uncompromised epidermis and pass through canals of hair follicles, in contrast to larger (500 nm) sized formulations. The appropriate size of nanoemulsions is recommended to be below 50 nm to achieve an efficient transdermal delivery [307]. Other important factors affecting transdermal transportation of nanoemulsions are the formulation composition and type of emulsion (*w*/*o* or *o*/*w*). The oil phase of the systems may be composed of fatty acids (e.g., oleic acid), esters of fatty acids and alcohols oils (isopropyl myristate/GRAS certified), triglycerides (triacetin/GRAS certified), as well nonpolar essential oils or lipid-soluble vitamins, among others [304,308]. Liu et al. [309] investigated the influence of different oil phases (eutectic mixture of menthol and camphor or isopropyl myristate) on transdermal delivery of glabridin-loaded nanoemulsions. According to the performed skin permeation studies, the formulation composed of binary eutectic mixture led to three times higher skin permeation of glabridin (compared to isopropyl myristate nanoemulsion), which was seven times higher than the isoflavane solution. Depending on the type of nanoemulsion, different permeation mechanisms were discussed. In the case of encapsulation of hydrophilic molecules in *w*/*o* nanoemulsions, transdermal transportation may be facilitated as a result of the solubilizing properties of the included surfactants on stratum corneum, and delivery via the pore pathway/hair follicles canals for large molecules. Regarding transdermal delivery of hydrophobic molecules from *o*/*w* nanoemulsions, active agent permeation may be achieved due to disruption of the *stratum corneum* (by creating permeable pathways as a result of fluidization of cell membranes and extracellular spaces), or improved permeation characteristics due to skin hydration (Figure 1) [307,310].

Various examples supporting the beneficial effects of lipid-based nanosystems concerning their improved physicochemical properties, or overcoming technological/biopharmaceutical limitations of different phenolic compounds, are presented in Table 2.

The beneficial effects observed after incorporating phenolic phytochemicals in lipid-based nanocarriers, such as improved solubility, stability and skin permeation/penetration, are a prerequisite for further research, development, and industrial application. In Table 3 are presented some cosmetic products based on lipid nanocarriers encapsulating various phenolic compounds. The observed favorable outcomes may be described as synergetic; on the one side they result from the well-known antioxidant, antiaging or skin whitening properties of the phenolic phytochemicals [330], and on the other side results may be attributed to the penetration-enhancing properties or occlusive effects of the lipid nanocarriers.

## 6. Conclusions

The numerous beneficial effects characteristic of phytochemicals with phenolic structures, such as anti-inflammatory, antioxidant, antiproliferative, and antiaging activities, determine their broad utilization potential in pharmaceutics and the cosmetic industry. However, these advantageous features cannot be fully exploited due to unfavorable physicochemical or pharmacokinetic characteristics (i.e., poor solubility, stability, bioavailability). Lipid-based nanosystems, such as liposomes, solid lipid nanoparticles, nanostructured lipid carriers and nanoemulsions, represent a successful approach to overcome these limitations and improve their dermal/transdermal delivery. This review thoroughly discusses the physicochemical properties and mechanism of actions of various classes of phenolic compounds regarding their dermal application. Examples of their incorporation in different lipid nanocarriers, as well a summary of the obtained results, are also provided. According to the data, encapsulation of phenolic compounds in lipid-based nanosystems for topical application leads to improved solubility, stability, skin permeation capability and therapeutic performance in general.

## Figures and Tables

**Table 1:** Physico-chemical characteristics of selected phenolic compounds.

Phenolic Class	Phenolic Subclass	Phenolic Compound	Molecular Weight (g/mol)	Partition Coefficient (Log P)	Solubility in Water at 25 °C (mg/mL)	Ionizable Groups with Corresponding pKa Values
Simple phenols and derivatives	Phenolic acids (hydroxybenzoic acid derivatives)	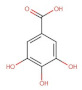 Gallic acid	170.12	−0.28 [208]	14.7 [209]	pKa^1^ = 4.51 pKa^2^ = 8.7 pKa^3^ = 11.4 pKa^4^ > 13 [210]
		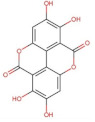 Ellagic acid (ellagitannin, dimer of gallic acid)	390.12	1.37 [211]	0.0097 [212,213] (at 37 °C)	pKa^1^ = 5.42 pKa^2^ = 6.76 [214]
	Phenolic acids (hydroxycinnamic acid derivatives)	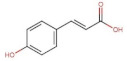 p-Coumaric acid	164.05	1.46 [215]	0.01 [216]	pKa^1^ = 4.92 pKa^2^ = 9.28 [217]
		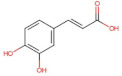 Caffeic acid	180.16	1.15 [218]	0.98 [209]	pKa^1^ = 4.83 pKa^2^ = 8.90 pKa^3^ = 10.28 [217]
		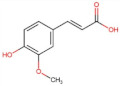 Ferulic acid	194.18	1.51 [219]	0.78 [209]	pKa^1^ = 4.66 pKa^2^ = 9.09 [217]
		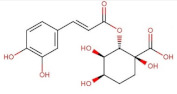 Chlorogenic acid	354.31	−0.75 [218]	3.44 * [220]	pKa^1^ = 3.50 pKa^2^ = 8.42 pKa^3^ = 11.00 [221]
	Other simple phenols	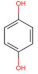 Hydroquinone	110.11	0.59 [222]	7.20 [223]	pKa^1^ = 9.85 pKa^2^ = 11.40 [224]
		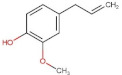 Eugenol	164.20	2.49 [225]	2.46 [226]	pKa = 10.19 [227]
Flavonoids	Flavones	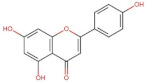 Apigenin	270.05	2.92 [228]	0.00135 [229]	pKa^1^ = 7.12 pKa^2^ = 8.10 [230]
		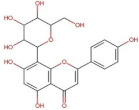 Vitexin (Apigenin-8-C-glucoside)	432.38	0.1 * [231]	0.0762 [232]	pKa^1^ = 6.27 * [233] **
		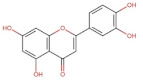 Luteolin	286.24	3.22 [228]	0.14 * [234]	pKa^1^ = 6.57 * [234] **
	Flavonols	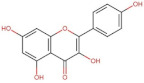 Kaemferol	286.23	3.11 [228]	0.113 [235] (at 30 °C)	pKa^1^ = 6.96 pKa^2^ = 8.78 pKa^3^ = 10.60 [236]
		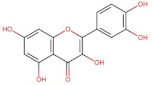 Quercetin	302.24	1.82 [228]	0.0004 [237] −0.002 [238]	pKa^1^ = 7.10 pKa^2^ = 9.09 pKa^3^ = 11.12 [236]
		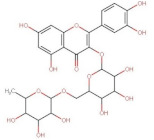 Rutin (Quercetin-3-O-rutinoside)	610.52	0.76 [22]	0.125 [239]	pKa^1^ = 2.92 pKa^2^ = 6.72 pKa^3^ = 8.26 pKa^4^ = 12.57 [240]
	Flavanones	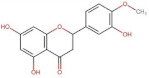 Hesperitin	302.27	2.9 [241]	0.01572 [241]	pKa^1^ = 7.55 * pKa^2^ = 8.50 * pKa^3^ = 9.65 *
		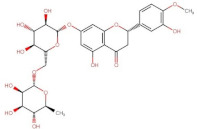 Hesperidin (Hesperitin- 7-(6-*O*-(alpha-l-rhamnopyranosyl)-beta-d-glucopyranosyl)	610.19	1.78 [242]	0.00495 [242]	pKa^1^ = 10.0 pKa^2^ > 11.5 [243]
	Flavan-3-ols	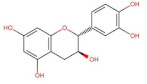 Catechin	290.26	0.41 [244]	7.66 [245]	pKa^1^ = 8.68 pKa^2^ = 9.70 pKa^3^ = 11.55 [236]
		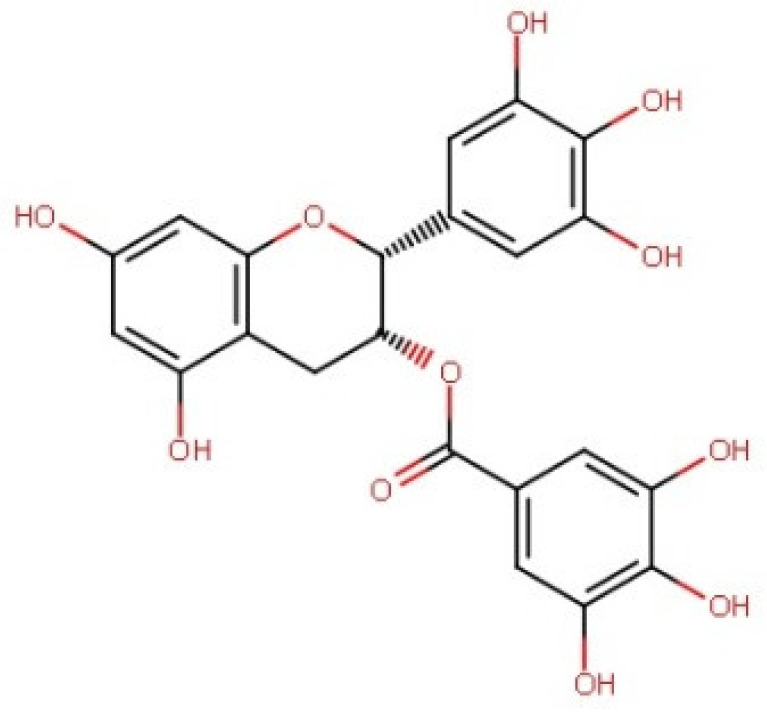 Epigallocatechin gallate	458.37	0.46 [246]	16.05 [247]	pKa^1^ = 7.75 pKa^2^ = 8.00 [248]
Curcuminoids		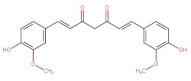 Curcumin (keto form)	368.38	3.0 [249]	0.0006 [250]	pKa^1^ = 7.7–8.5 pKa^2^ = 8.5–10.4 pKa^3^ = 9.5–10.7 [249]
Stilbenes		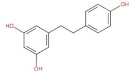 Resveratrol	228.25	3.09 * [251]	0.05 [252]	pKa^1^ = 8.8 pKa^2^ = 9.8 pKa^3^ = 11.4 [253]
Anthraquinones		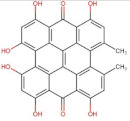 Hypericin	504.44	3.43 [254]	Practically insoluble in water [255] **	pKa^1^ = 2.00 pKa^2^ = 11.00 [256]
Phloroglucinols		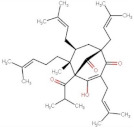 Hyperforin	536.78	13.17 [257]	2.34 × 10^−12^ [257]	pKa = 6.32 * [258]

* calculated value; ** information (or further information) was not found.

**Table 2:** Phenolic compounds, encapsulated in lipid-based nanosystems for dermal. application.

Main Class	Active Agent	Technological/Biopharmaceutical Issue	Lipid-Based Nanosystem/ Lipid Carrier	Obtained Results	References
Simple phenols and derivatives	Hydroquinone	Tendency to oxidation; hydrophilic structure hindering its topical application; side-effects due to systemic absorption	SLNs/Precirol^®^ ATO5	High hydroquinone encapsulation (app.90%); long-standing physico-chemical stability; enhanced skin accumulation	[311]
	Arbutin	Highly hydrophilic compound; limited skin permeation	Liposomes/ Soybean phosphatidylcholine; cholesterol	Increased skin whitening efficacy; Improved arbutin deposition in epidermis/dermis	[312,313]
	Thymoquinone (benzo-quinone)	Thermo/photosensitivity; hydrophobicity	Liposomes/ Phospholipon 90H^®^; cholesterol	Improved anti-inflammatory action compared to plain solution; enhanced stability against degradation; increased skin permeation	[314]
	Protocatechuic acid; Ethyl protocatechuate (phenolic acid and derivative)	Sparingly hydrosolubilty (1:50); skin irritating properties; photosensitivity	SLNs/ Precirol ATO^®^5; NLCs/ Miglyol^®^810 N: Precirol ATO^®^5 3:7	NLCs are the superior nanosystem concerning PDI and cell viability results compared to SLNs; minimized skin irritation potential of protocatechuic acid; ensured UVB protection; controlled release profile of phenolic acids without systemic exposure	[315,316]
	Ferulic acid (phenolic acid)	Poor water solubility; low stability	Nanoemulsion/ Isostearyl isosearate	Improved solubility and permeability of ferulic acid; significant antioxidant effect	[317]
	Caffeic acid (hydroxyl-cinnamic acid)	Limited skin permeation	Liposomes/ egg phosphatidyl-choline; cholesterol	High entrapment efficiency values (70%); improved penetration compared to free caffeic acid; preserved antioxidant activity	[318]
	Eugenol (Clove oil); (phenylpropene)	Predisposition to oxidation	SLNs/Stearic acid, Compritol^®^	Development of eugenol loaded SLNs incorporated in carbopol hydrogel; improved eugenol deposition in epidermis, compared to reference formulation; achieved controlled release profile; sufficient occlusive properties	[319]
Flavanoids	Naringin (flavanone)	Limited aqueous solubility; poor oral bioavailability	Liposomes/ Epikuron-200; cholesterol, Tween 80	Improved skin deposition; high encapsulation efficiency (99%); very good physical stability	[320]
	Hesperidin (flavanone glycoside)	Poor aqueous solubility and bioavailability	NLCs/ Cupuaçu butter, buriti oil	High encapsulation efficiency (96%); sufficient physical stability; noncytotoxic effect on melanoma cell lines	[321]
	Isoflavone-aglycon-rich fraction (genistein, daidzein, glycitein)	Limited aqueous solubility	Nanoemulsion/ Egg lecithin (Lipoid E-80^®^), medium-chain triglycerides	Improved aqueous solubility; development of nanoemulsion/hydrogel; achieved active agent deposition in stratum corneum, epidermis and dermis (highest)	[322]
Anthraquinones	Aloe -emodin	Hydrophobic compound, crystallizes in water	Liposomes/ Hydrogenated soybean phosphatidyl-choline, cholesterol	Improved skin permeation; low cytotoxicity	[323]
Naphthoquinones	Vitamin K1 (phylloquinone)	Highly lipophilic compound; photosensitivity	Liposomes/ Soy phosphatidyl-choline, α-tocopherol	Elaboration of liposomal aqueous dispersion applied by nebulization; Improved aqueous solubility; increased deposition in epidermis and dermis compared to ointment formulation	[324]
Xanthones	Mangiferin	Poor water solubility (0.111 mg/mL); low bioavailability	Nanoemulsion/ Almond oil, Lipoid ^®^S75	Enhanced permeation; anti-inflammatory effect; reduced edema and leukocyte infiltration	[325]
Stilbenes	Resveratrol	Low aqueous solubility; photosensitivity	SLNs/ Compritol 888 ATO NLCs/ Compritol 888 ATO; Miglyol oil	*Resveratrol* loaded NLCs were superior to SLNs with respect to entrapment efficiency values, skin penetration, accumulation in dermis	[326,327]
	Pterostilbene	None; favorable characteristics compared to *resveratrol* (i.e., increased lipophilicity, membrane permeability and bioavailability)	Liposomes/ Lecithin	Effectively prevents UVB-radiation induced skin carcinogenesis in mice	[328]
Tannins	Ellagic acid	Low aqueous solubility and permeability	NLCs/ Tristearin, Miglyol oil	Improved solubility; high antioxidant activity	[329]

**Table 3:** Cosmetic products containing phenolic phytochemicals formulated via nanotechnological approach.

Brand	Product	Phenolic Compounds	Lipid-Based Nanosystem	Benefits
Sesderma [331]	Sodyses Repair gel	Resveratrol, quercetin	Liposomes	Supports healing process and proper skin recovery, reduced scar tissue formation
	Factor G Renew Rejuvenating serum	Quercetin, pterostilbene	Liposomes	Promotes cell regeneration, increased collagen and elastin synthesis antiwrinkle effect
	Hidroquin Whitening gel	Ferulic acid, Arbutin	Liposomes	Skin whitening properties; prevention and treatment of different skin imperfections
	Reti Age Eye contour gel	Pterostilbene	Liposomes	Provides skin rejuvenation
	Kojicol Plus (+Kojic acid) Skin lightener cream	α-Arbutin,	Liposomes	Skin whitening properties
M.Y.R. [332]	Curcumin liposome Melasma and acne cream	Curcumin	Liposomes	Diminishes melasma patches, freckles, dark spots, acne scars Antiaging effect
Vitacos [333]	NanoVital Vitanics Whitening essence	Arbutin	Nanoemulsion	Moisturizing and skin lightening properties
Dr. Theiss Medipharma Cosme-tics [298]	Olivenöl Anti Falten Pflege-konzentrat	Olea europaea oil	NLCs	Promotes cell regeneration and skin rejuvenation

## Data Availability

Data sharing not applicable.
